# A new, fast algorithm for detecting protein coevolution using maximum compatible cliques

**DOI:** 10.1186/1748-7188-6-17

**Published:** 2011-06-14

**Authors:** Alex Rodionov, Alexandr Bezginov, Jonathan Rose, Elisabeth RM Tillier

**Affiliations:** 1 The Edward S. Rogers Sr. Department of Electrical and Computer Engineering, University of Toronto, Toronto, Canada; 2 Department of Medical Biophysics, University of Toronto, Toronto, Canada; 3 Ontario Cancer Institute, University Health Network, 101 College Street., Toronto, M5G 1L7, Canada

## Abstract

**Background:**

The MatrixMatchMaker algorithm was recently introduced to detect the similarity between phylogenetic trees and thus the coevolution between proteins. MMM finds the largest common submatrices between pairs of phylogenetic distance matrices, and has numerous advantages over existing methods of coevolution detection. However, these advantages came at the cost of a very long execution time.

**Results:**

In this paper, we show that the problem of finding the maximum submatrix reduces to a multiple maximum clique subproblem on a graph of protein pairs. This allowed us to develop a new algorithm and program implementation, MMMvII, which achieved more than 600× speedup with comparable accuracy to the original MMM.

**Conclusions:**

MMMvII will thus allow for more more extensive and intricate analyses of coevolution.

**Availability:**

An implementation of the MMMvII algorithm is available at: http://www.uhnresearch.ca/labs/tillier/MMMWEBvII/MMMWEBvII.php

## Background

An important problem in evolutionary biology is the comparison of phylogenetic trees [1]. Tree comparisons have been performed to establish the accuracy of phylogeny building methods [2-4], to determine inconsistencies between the phylogenetic history of different genes and thus determine horizontal transfer of genes between species [5,6], to find orthologous genes [4] and to identify genes that coevolve [7,8]. Some classical methods only compare tree topologies and the problem has been to identify an appropriate distance measure which describes the branch rearrangements to transform one tree into another. However most applications, which aim to find correlated rates of evolution, require the comparison measure to also consider differences in branch lengths between the trees compared.

In the case of determining coevolution, where it is required that two independent genes have correlated rates of evolution, the consideration of branch lengths is critical. Proteins that interact with one another affect each others' rate of evolution such that these are more likely to evolve with correlated rates -- a process known as *coevolution*. Proteins that coevolve have similar evolutionary histories, in terms of both the tree topology and correlated branch lengths, and this can be leveraged to predict which proteins interact.

The detection of coevolution thus requires gauging the similarity of two phylogenetic histories. A number of methods have been developed to detect coevolution, such as the *mirror tree *[7,9-12] approach. This technique compares the evolutionary histories of two families of homologous proteins. However, the phylogenetic trees are not directly compared. Rather, the evolutionary history of each family is quantified by calculating a phylogenetic distance matrix, which determines the genetic distance between every pair of proteins in the family. The distances are determined from the multiple sequence alignment (MSA) of the sequences. Interacting protein partners between the two families are identified by maximizing the statistical correlation between their distance matrices.

A distance matrix is an indirect representation of a family's phylogenetic tree. Other approaches compare these trees directly [8] and our own earlier program Codep [13] compares the multiple sequence alignments to measure the coevolution signal.

The drawbacks of these previous approaches include the requirement that the two protein families be the same size, such that the composition of the protein families must be pre-processed beforehand either by careful screening [7] or by random sampling [9,13]. This means that the inclusion of paralogs (which lead to multiple possibilities for interaction partners) is not handled well. The methods also make the assumption that the protein families have coevolved throughout the entire evolutionary history of the sequences considered.

**Figure 1:**
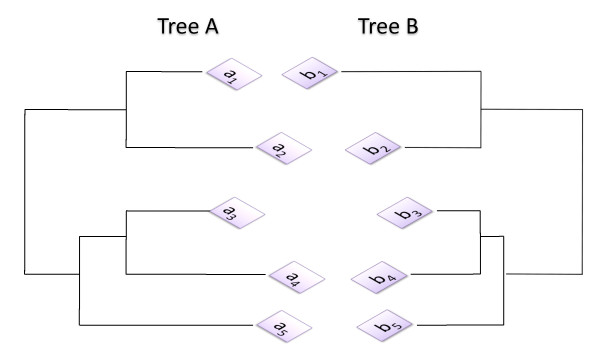
**Example of coevolution of two protein families**. Although MMM makes use of distance matrices, we can illustrate the solution sought by considering the phylogenetic trees of two protein families A and B. The sequences from a3, a4 and a5 would match with the corresponding proteins b3, b4 and b5 by the MMM algorithm because the subtree of those sequences in A is only different from the corresponding subtree in B by a scaling factor in the branch lengths. With a strict tolerance, sequences from species 1 and 2 would not contribute to the match, as the relative branch lengths to the other sequences are very different.

We recently proposed MatrixMatchMaker (MMM) [14,15], an alternative algorithm that addresses these issues. As in the mirror tree approaches, MMM uses the distance matrices of the protein families as input. Instead of using statistical correlation to detect coevolution, MMM searches for pairs of submatrices that are similar (one being a scaled version of the other) within a tolerance. These similar submatrices represent similar phylogenetic subtrees, and identify the proteins involved in similar parts of the two families' evolutionary histories (Figure 1).

The advantages of the MMM approach are that:

1. The initial distance matrices can be of different sizes, allowing protein families with unequal numbers of homologues to be interrogated for coevolution.

2. The algorithm is able to discover coevolution in any subset of the evolutionary history of the proteins.

3. All possible solutions are returned, allowing coevolution between specific or multiple paralogues to be discovered.

The approach was shown to increase the accuracy over mirror tree approaches. This is partly due to the reduced sensitivity of MMM to artifacts stemming from the assembly of protein families which can strongly affect the Pearson correlation score [14] that mirror tree methods use to correlate two distance matrices. MMM is also less sensitive to false positive determination of coevolution due to long internal branches shared between two trees with highly divergent species. This strong phylogenetic signal will result in a large Pearson correlation of the distance matrices, but is not strong evidence for functional coevolution. Our approach requires all distances to match in the solution which results in higher accuracy.

MMM thus is a better method for predicting coevolution than the mirror tree approach, however this comes at the cost of having to solve a much more computationally demanding combinatorial problem; because all submatrices of each family must be considered, the search space is exponential in size.

In this paper we present a novel approach for finding the set of largest similar submatrices. This new algorithm (MMMvII) solves the problem of finding the maximum submatrices exactly, and is rendered much faster than MMM by expressing the problem as a series of maximum clique problems, and leveraging existing efficient techniques to solve them.

## Methods

To compare the accuracy and performance of MMMvII to those of the original MMM an evaluation data set comprised of pairs of distance matrices was compiled using the OMA database [16]http://www.omabrowser.com.

We obtained all eukaryotic clusters from the OMA version dated October 2010 and re-clustered them via CD-HIT [17]http://bioinformatics.ljcrf.edu/cd-hi/ at an 80% sequence identity threshold in order to merge the paralogous clusters, possibly resulting in species being represented multiple times. The biological reasonableness of this clustering approach for predicting protein-protein interactions has not yet been validated and will be investigated elsewhere. Our purpose here was to create a large dataset of difficult problems on which we could compare the performance of the algorithms.

Multiple sequence alignments (MSA) were obtained on each resulting cluster using MAFFT 6.716b [18]http://mafft.cbrc.jp/alignment/software/. Next, the distance matrices for each alignment were created with Protdist 3.69 [19]http://evolution.genetics.washington.edu/phylip/progs.data.prot.html modified to allow selenocysteine and pyrolysine amino acids and for identical sequences to have a distance of 0.0 (Protdist originally sets these to 0.00001).

Finally, we compiled a set including all-by-all pairwise combinations of matrices that shared at least 30 species in common (17,969,452 pairs).

All MMM experiments described here were conducted on a cluster of 72 Intel Xeon processors at 3.06 GHz with 2 GB of RAM available to each.

Initially, the entire data set was processed using the original MMM with the threshold parameter *α *set to 0.1. However, during the allotted time (2 months) it was able to complete the analysis of only 819,014 pairs. As a result, all ensuing comparisons with MMMvII were performed only on these pairs.

Due to the increasing relative error for shorter times, only the 26368 pairs for which the time of MMM runs was at least 5 seconds were considered (for the excluded pairs the MMMvII time never exceeded 0.15 seconds).

The accuracy of prediction of known protein-protein interactions was compared for the two algorithms as in [14]. Instead of using multiple individual databases of protein interactions, we used the iRefIndex database [20]http://irefindex.uio.no, since it comprehensively compiles protein interaction data from multiple public databases in a non-redundant manner.

## Results and Discussion

### Problem Formulation

Given two families of homologous proteins **A **and **B**, we would like to predict the likelihood of interaction between them by detecting the number of coevolving **A**-to-**B **protein pairs. Let **A **= {*a*_1_, *a*_2_, ..., *a_n_*} and **B **= {*b*_1_, *b*_2_, ..., *b_m_*} be the two protein families in question, which can, in general, be of unequal size.

Consider a set **M **of *k *protein pairs , which pairs up *k *proteins from **A **with *k *proteins from **B **in a one-to-one fashion. If both proteins in every pair in M have similar evolutionary histories, then we say that **M **forms a *match of size k*. The size of the largest possible match given **A **and **B **indicates the amount of coevolution between the families.

The set of pairwise phylogenetic distances between all the **A **proteins in **M **can be thought of as representing the evolutionary history of those proteins, via sums of branch lengths in an implied phylogenetic tree. A set of **A**-to-**B **protein pairings also implicitly pairs up the associated distances between the **A **proteins with the distances between the **B **proteins. If the distances between all the **B **proteins in **M **are equal to that of their paired **A **distances multiplied by a common scale factor, then the two histories are considered similar and **M **will be a match. This condition will now be further defined with more notational precision.

Let *d*(*p*, *q*) be the phylogenetic distance between any two proteins *p *and *q *from the same family. Given two **A**-to-**B **protein pairs (*a_u_*, *b_x_*) and (*a_v_*, *b_y_*), define the *ratio of paired distances *(RPD) for those two pairs as *R*(*a_u_*, *b_x_*)(*a_v_*, *b_y_*) = *d*(*a_u_*, *a_v_*)/*d*(*b_x_*, *b_y_*). In the ideal case, if **M **is a match of size k then all *k*-choose-2 RPDs would have the same value, indicating that the **A **distances are a scaled copy of their paired **B **distances. However, we must add some tolerance in order to accept matches that deviate slightly from this ideal scaling.

This tolerance is controlled by a parameter *α *∈ [0, 1], with 0 requiring all RPDs be exactly the same value and 1 placing no restrictions on values amongst RPDs. Using this parameter, we define that two RPDs *R*_1 _and *R*_2 _are *compatible *if:

Note that if *R*_1 _is compatible with *R*_2 _then *R*_2 _is also compatible with *R*_1_. Using this definition of compatibility, we can now more precisely state that **M **forms a match if every pair of RPDs between its *k *protein pairs is compatible. Additionally, when these are specified, we can only allow proteins from the same species to be paired within a match. Only nonzero phylogenetic distances are considered.

**Figure 2:**
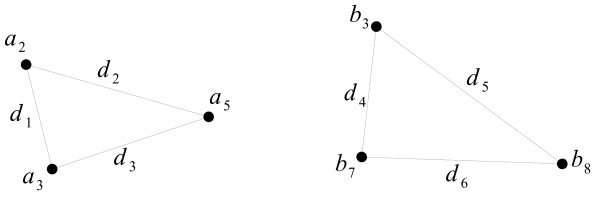
**Example showing two triplets of proteins and the phylogenetic distances between them**.

As an example, consider two triplets of proteins: {*a*_2_, *a*_3_, *a*_5_} ⊆ **A **and {*b*_3_, *b*_7_, *b*_8_} ⊆ **B**, with corresponding phylogenetic distances *d*_1 _through *d*_6_, as depicted in Figure 2. If *d*_1_/*d*_4 _≈ *d*_2_/*d*_5 _≈ *d*_3_/*d*_6_, under a given *α*, then we consider the set of protein pairs {(*a*_2_, *b*_3_), (*a*_3_, *b*_7_), (*a*_5_, *b*_8_)} to form a match of size 3, with each pair representing two coevolving proteins.

**Figure 3:**
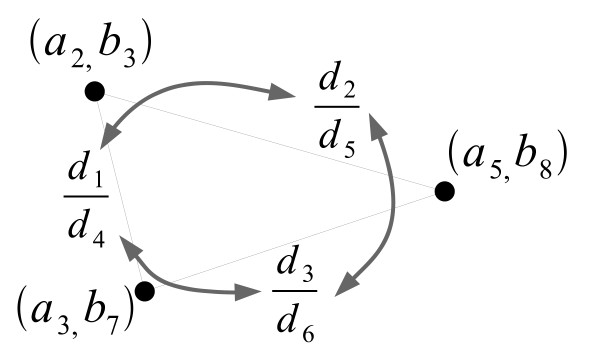
**Graph-based representation of the example in Figure 2**. Vertices represent protein pairs, and edges are labeled with the RPD for the pairs they connect. Arrows indicate the pairs of RPDs that must be compatible in order to satisfy the conditions for forming a match.

The discussion so far has concerned the determination of whether or not some set of protein pairs forms a match. We can now use a similar representation as in Figure 3 to represent the original coevolution problem: given the input families **A **and **B**, find the size of the largest possible match.

**Figure 4:**
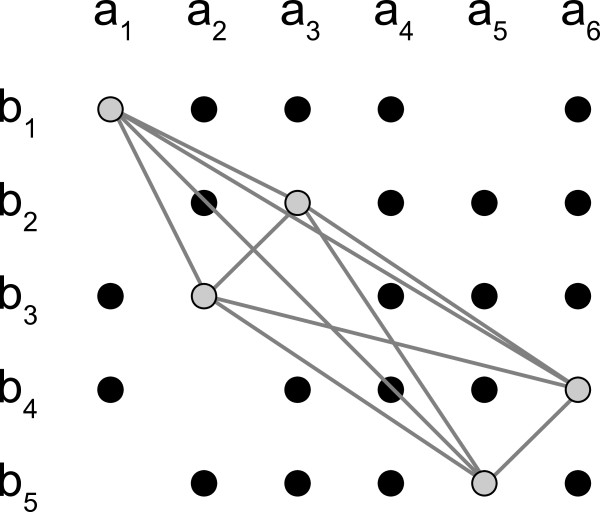
**Example compatibility graph**. Example compatibility graph for two protein families **A **= {*a*_1 _... *a*_6_} and **B **= {*b*_1 _... *b*_5_}. Circles are vertices representing an *a_i _*to *b_j _*protein pair from matching species. The grey vertices are pairs that will form a match of size 5 if all 10 connecting edges (also in grey) are compatible with each other. Edges between vertices not included in the maximum clique are omitted for clarity.

Here, we introduce the concept of a *compatibility graph*, an example of which is shown in Figure 4. In this graph, there are up to *n *× *m *vertices, representing all possible **A**-to-**B **protein pairs. Edges exist between every two vertices that could ever appear together in any match, and are labeled with the corresponding RPD for that pair of vertices. This connectivity results in a very dense graph, with an edge between any two vertices except when the corresponding RPD has a zero distance in either its numerator or denominator. As a result, no edges exist between any two vertices in the same row or column, because the **A **or **B **distances within the corresponding RPD would be zero.

For an edge *e *connecting two vertices *u *and *v *in the compatibility graph, *R*(*e*) and *R*(*u*)(*v*) both equivalently refer to the RPD between the two protein pairs represented by *u *and *v*. Furthermore, two edges are said to be compatible if their RPDs are compatible.

In graph terminology, a set of vertices forms a *clique *if every pair of vertices in the set is connected by an edge. Under the graph-based representation of the coevolution problem, matches are cliques in the compatibility graph whose edges are all pairwise compatible. Therefore the solution to the problem of finding the largest match size is to find the size of the maximum cliques of the compatibility graph whose edges are also all pairwise compatible. This approach solves the coevolution problem exactly.

### Algorithm

In this section, we present the MMMvII algorithm that solves the problem posed above.

The input to the algorithm is the compatibility graph **G **= (**V**,**E**) constructed from two protein families **A **and **B**, along with a tolerance *α *∈ [0, 1]. The output will be the set of all the matches of largest size, with each match representing one possible configuration of coevolving protein pairs.

Before describing the algorithm, we require a new definition. Given the tolerance *α*, an RPD *R*_1 _is *forward-compatible *with *R*_2 _if:

This is similar to the definition of compatibility between two RPDs, except "one-sided", such that if *R*_1 _is forward-compatible with *R*_2 _then *R*_2 _cannot be forward-compatible with *R*_1 _unless *R*_1 _= *R*_2_. Two edges are forward-compatible if their RPDs are forward-compatible. In any given set of edges, there exists at least one edge with the smallest RPD value among all of them, called the *edge of minimum RPD *for that set. A result, which can be easily derived, is that if every edge in a set is forward-compatible with the set's edge of minimum RPD, then every pair of edges is mutually compatible (assuming the same value of *α*).

In the algorithm to be described, we will use this result to help find the largest matches. For each edge in the compatibility graph, we will assume that that edge is the edge of minimum RPD of some set of edges, and then "work backwards" to find that set. All the edges in each set are then guaranteed to be pairwise compatible. At that point, we find the maximum cliques of each set, which form matches of maximum size.

**Figure 5:**
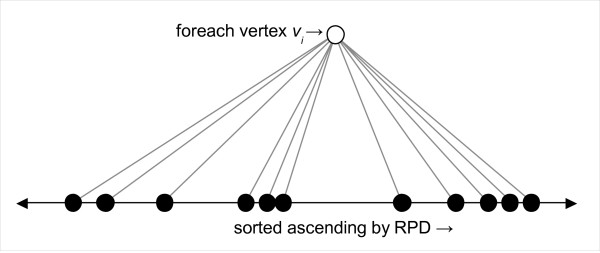
**Step 1**. After choosing vertex *v_i _*(white dot), its neighbours (black dots) are sorted in ascending order of the RPDs of their edges to *v_i_*.

The outer loop of the algorithm iterates over all vertices *v_i _*in **G**. For each *v_i_*, we build a list of its neighbour vertices, which are sorted in ascending order of the RPDs of their edges to *v_i _*(Figure 5).

**Figure 6:**
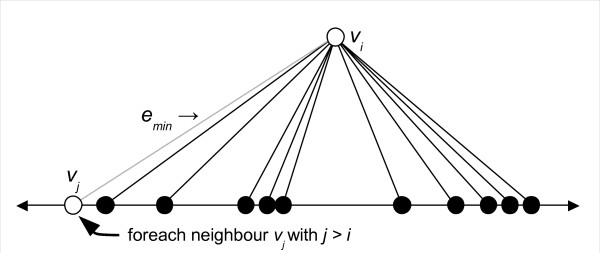
**Step 2**. After choosing a vertex *v_j _*from the sorted neighbour list, the edge from *v_i _*to *v_j _*is declared to be *e_min _*- the current edge of minimum RPD.

After choosing *v_i_*, another loop iterates over all vertices *v_j _*in the sorted neighbour list. We consider only those *v_j _*where *j *>*i*, in order to avoid visiting the edges in **G **twice. The edge between *v_i _*and *v_j _*is denoted *e_min_*, which will be the edge of minimum RPD for the remainder of this inner *v_j _*loop. This step of the algorithm is shown in Figure 6.

**Figure 7:**
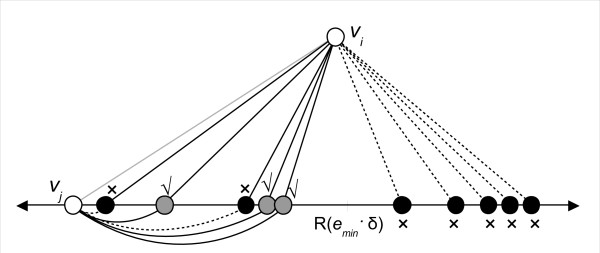
**Step 3**. Vertices ahead of *v_j _*in the sorted neighbour list are found whose edges to both *v_i _*and *v_j _*are forward-compatible with *e_min _*(solid edges). These vertices, shown in grey with a check beside them, form the vertex set of the subgraph **H**. Vertices in the sorted list which fail this test, due to the presence of one or more non-forward-compatible edges (dashed) are indicated with an X. Vertices to the right of the sorted list automatically fail the test - their edges to *v_i _*have RPDs greater than *R*(*e_min_*)·*δ *and therefore are not compatible with *e_min _*due to the sorting of RPDs performed earlier.

The next step, shown in Figure 7, builds the vertex set of a subgraph of **G **that we call **H**. It walks through the sorted neighbour list, considering all vertices *v_k _*ahead of *v_j _*in the list for inclusion in **H**. Each *v_k _*is tested to see whether *both *its edges to *v_i _*and *v_j _*are forward-compatible with *e_min_*. This condition is necessary for *v_k _*to be part of the same match as *v_i _*and *v_j _*. Note that because *v_i_*'s neighbour list is sorted by RPD of the edge to *v_i_*, the walking of the neighbour list in this step can be terminated early once one *v_k _*is found whose edge to *v_i _*is no longer forward-compatible with *e_min_*. Finally, if *R*(*v_i_*)(*v_k_*) or *R*(*v_j_*)(*v_k_*) are equal to *R*(*e_min_*), then *v_k _*is only included in **H **if *k *>*i*. This extra check prevents duplication of results in later choices of *v_i_*.

Having created the vertex set of **H**, we next form the edge set. An edge in **H **exists between every pair of distinct vertices (*v_x_*, *v_y_*) where *R*(*v_x_*)(*v_y_*) is forward-compatible with R(emin). However, if *R*(*v_x_*)(*v_y_*) = *R*(*e_min_*), then we also require that the indices *x *and *y *must both be greater than the index *i *of *v_i _*for an edge to exist. This prevents the algorithm from duplicating results in future iterations of *v_i_*.

With **H **formed, its maximum cliques are found. For the purposes of our algorithm, any exact (optimal) maximum clique finding algorithm will suffice. We used Östergård's algorithm [21], modified to give all cliques of maximum size instead of just exiting after one. However, if one only wishes to find the size of the largest matches in **G **along with just one of the matches (instead of all of them), then this modification is not necessary and faster performance can be obtained. We implemented this option as well ('maxtrees = 1' option).

**Figure 8:**
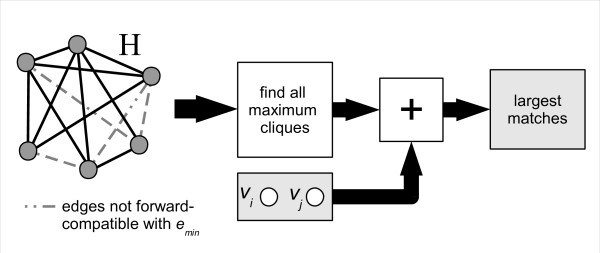
**Step 4**. The edge set of **H **is constructed, and includes all possible edges between the vertices of **H **that are forward-compatible with the current *e_min_*. After finding all the maximum cliques of **H**, *v_i _*and *v_j _*are appended to each maximum clique, and considered in the set of largest matches for the entire problem.

Each of the maximum cliques returned is a match, since all the edges in **H **were made to be mutually compatible by construction. Vertices *v_i _*and *v_j _*are also added to every returned match. This is possible because there exist edges from every clique member to *v_i _*and *v_j _*, and those edges are compatible with the rest of the match's edges - again true by construction of **H**. This concludes the final series of steps, starting from the construction of **H**'s edge set, shown in Figure 8.

This set of matches represents the largest matches possible in **G **that are constrained to have *v_i _*and *v_j _*as members. The final step is then to continue iterating over all remaining *v_i _*and *v_j_*, collecting the matches from each iteration and keeping only the globally largest ones, which yield the solution to the entire problem. If during any choice of *v_j _*it can be guaranteed that the matches that will result from this iteration are to be *smaller *than the current best match size, then the current *v_j _*can be abandoned. For example, one can count the number of vertices in **H **after Step 3, and if this number plus two (for *v_i _*and *v_j_*) is smaller than the current best match size, then it is pointless to proceed further with that **H**. Some tighter bounds are described in [22].

As a note on algorithm complexity, there are *O*(|V|^2^) edges in G, and each edge creates an instance of a maximum clique problem, which is a well-studied NP-hard problem [23]. Since MMMvII requires exact solutions to these maximum clique subproblems, its worst-case time complexity is exponential. However, the actual performance of an efficient maximum clique algorithm depends on the structure of the input graph. Pseudocode for the algorithms are given in Additional file 1.

MMMvII still solves (with minor differences) the same problem as MMM in an exact manner, meaning both algorithms must have NP-hard worst-case characteristics and could potentially perform equally poorly. Therefore, MMMvII's significantly better measured performance compared to MMM (which we will show) implies that, in practice, the maximum clique problems generated by MMMvII do not actually exhibit the worst-case exponential behaviour.

### Differences with MMM

The original MMM algorithm iterates through all possible matches of size 3 in an exhaustive fashion, ordered by protein indices within A and B, with an early exit if the number of remaining proteins in the loop cannot exceed the size of the largest matches found so far. A recursive subroutine attempts to expand an existing match by including a new pair of proteins. For each protein pair, it must be determined whether or not its inclusion in the existing match results in a new, larger match. In this algorithm, this step is done by testing for all matches of size 3 that are created by the inclusion of the new protein pair. Each triplet to be checked contains the new protein pair and two other protein pairs in the existing match. The mutual compatibility test must pass for all such triplets. If the addition of a protein pair successfully creates a new, larger match, a recursive call is made to further expand the match until all protein pairs have been iterated through. At each level of recursion, the list of matches is updated if the current match matches or exceeds the current record for the largest match. Only the largest matches are kept, which become the output of the algorithm.

This triplet-based check is not an exact test of compatibility within the new match, and is an approximate heuristic designed to be fast rather than exact. The rationale behind designing this algorithm was that a full compatibility test of the new match would require every ratio of paired distance to be checked against every other ratio of paired distances -- an operation whose time complexity scales to the fourth power of the number of protein pairs in the match. This approximate triplet-based compatibility check only scales to the second power. As such, the original approach may give false positives and not exactly solve the coevolution problem.

While MMM and MMMvII both solve the same fundamental coevolution problem, they diverge in their criteria for deciding whether or not a given set of A-to-B protein pairs have similar evolutionary histories. Thus, the results from both algorithms may, in principle, differ when given the same inputs. Despite MMMvII's new graph-based view of the coevolution problem (which MMM lacks), the different coevolution criteria can still be explained intuitively using MMMvII terminology. Given a set of 3 protein pairs, both MMM and MMMvII will always agree on whether or not that set forms a match - their behavior for triplets of pairs is identical. However, for a set of *k *> 3 protein pairs, MMM takes every possible triplet of pairs from that set and tests if it forms a match of size 3. This is in contrast with MMMvII which provides an elementary definition for matches of size greater than 3 that does not recursively depend on the definition of a match of size 3. The result is that for all *k *≥ 3, an MMMvII match also forms an MMM match. This is because any subset of protein pairs of an MMMvII match also forms an MMMvII match. Since subsets of size 3 are treated identically by MMM and MMMvII, all triplets of an MMMvII match will be MMM matches, and thus the larger match will be an MMM match as well. This relationship does not hold in general in the opposite direction - an MMM match of size *k *> 3 is not necessarily an MMMvII match. Hence, we say that MMMvII has a stricter definition of a match than MMM, and will return a subset of its results. However, we will present results that show that, in practice, these different criteria result in negligible differences in terms of the maximum submatrix size obtained between MMM and MMMvII.

### Performance

**Figure 9:**
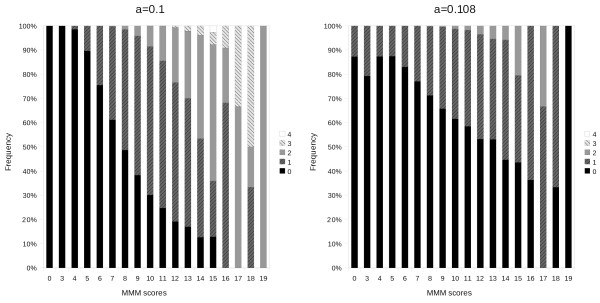
**Accuracy**. Plotted is the frequency of the absolute difference between the size of the largest submatrix returned by MMMvII (the MMM score) versus that returned by the original MMM algorithm. The results show that the scores produced by MMMvII do not differ substantially from those of the original MMM, particularly when the tolerance is slightly increased to compensate for the increased strictness of the MMMvII algorithm.

The implementation of stricter submatrix matching is the only difference between MMMvII and the original MMM algorithm which could affect the size of the resulting submatrices (the MMM score), which we would expect to be lower at the same tolerance parameter *α*. More dangerously, this effect would be most profound in higher scores, which in turn are the most important for the data analysis. Thus, in order to account for any effects of this systematic difference on either accuracy and/or performance, a series of MMMvII runs were performed to identify the tolerance parameter *α *which would make the total sum of squared scores as close as possible to the original distribution of scores. Since an *α *= 0.1 was previously empirically determined to work well for the prediction of protein-protein interactions [14,15], we found the slightly more relaxed tolerance of *α *= 0.108 was required for MMMvII. Indeed, the absolute differences of scores produced by MMMvII at *α *= 0.1 can be as high as 4, whereas at *α *= 0.108 the differences are generally lower, and never exceed 2 (Figure 9). These differences in score were too slight to produce any difference in the overall accuracy of protein interaction predictions.

**Figure 10:**
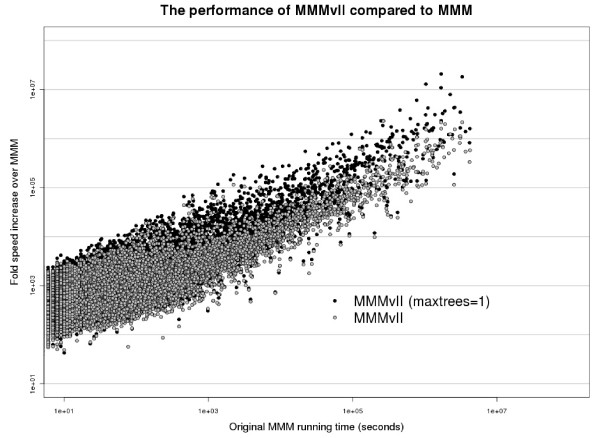
**Speedup of MMMvII over original MMM**.

The recursive nature of the MMM algorithm is such that solutions with higher score will take more time to compute than small solutions, and MMMvII would be expected to be faster due to the stricter matching requirement and smaller scores. We thus compared two programs at their equivalent tolerance values. The speedups (*oldtime*/*newtime*) of MMMvII (*α *= 0.108) against the old MMM algorithm (*α *= 0.1) ranged from 42× to 2, 198, 568× in individual pairs, with a geometric mean speedup of 639× and total run time for the whole dataset being reduced by 41, 105×. In comparison, for MMMvII executed with *α*=0.1, the geometric mean speedup was even higher at 710×, and the total total run speedup at 47, 010×. Therefore, the stricter matching implemented in the MMMvII algorithm does indeed result in faster running times. However, this effect is only moderate, and when using the adjusted tolerance parameter, MMMvII is still much faster than the original implementation. Importantly, the higher speedup values corresponded to the pairs that had required very long execution times when using the original MMM program (Figure 10). Thus, the performance improvement of the MMMvII over the original MMM has a drastic effect on reducing the total running time.

An additional performance gain can be achieved by allowing the program to avoid returning multiple solutions and instead return only the MMM score and a single submatrix solution ('maxtrees = 1' option). This feature is useful for large scale applications, where coevolving pairs with high scores need to be identified rapidly, without consideration of all the possible solutions. While keeping the tolerance at *α *= 0.108, this optimization gave an even higher speedup for both geometric mean: 876×, and the total running time: 64, 653× (Figure 10).

## Conclusion

We have presented an improved algorithm for detecting coevolution between clusters of homologous protein sequences. The MMMvII algorithm reformulates MMM's original method of finding maximum common submatrices into a graph-theoretical problem of finding maximum similar cliques. While still being a recursive algorithm, the MMMvII algorithm efficiently culls the remaining search space at every recursion level, and thus gave an average speedup of over 600 times for the dataset we used. MMMvII retains the original intent of MMM, that is to find the largest submatrix match within a tolerance but does so more exactly by enforcing the strict adherence to this tolerance, removing the approximations made by the original MMM.

The faster MMMvII algorithm permits the rapid analysis of larger protein families incorporating more information from the vast amount of sequence data being generated. The very large dataset we assembled was incompletely run with MMM in over two months. Those runs it did complete (5.5% of the entire dataset), were run in just half an hour with the new program. MMMvII also could manage the entire dataset in 67 hours on the same hardware. MMMvII thus allows the investigation of much larger datasets, and those where the analysis includes paralogous families. Although MMM did allow for the consideration of paralogous families to detect multiple interactions, the original approach was too slow to be practicable. MMMvII will thus allow for more extensive and intricate analyses of coevolution. As a general tool for measuring the similarity between phylogenetic trees and distance matrices, the MMM algorithm could also be used in other areas in comparative genomics and computational sequence analysis.

## Competing interests

The authors declare that they have no competing interests.

## Authors' contributions

AR developed, designed and programmed the algorithm under the supervision of JR and ERMT. AB prepared the sequence data for analysis, performed the accuracy and speed tests and analyzed the results with ERMT. All authors contributed to the writing and editing of the manuscript, and all authors read and approved the final manuscript.

## Supplementary Material

Additional file 1 **Pseudocode for MMMvII**. Pseudocode for the MMMvII algorithm, including the modified Östergård procedures. Click here for file
