# Supplementary material for: A new, fast algorithm for detecting protein coevolution using maximum compatible cliques
Source: Algorithms Mol Biol. 2011 Jun 14;6:17. doi: 10.1186/1748-7188-6-17 (PMC3130660; doi:10.1186/1748-7188-6-17)
Supplement: Additional file 1 — Pseudocode for MMMvII. Pseudocode for the MMMvII algorithm, including the modified Östergård procedures. [file 1748-7188-6-17-S1.PDF]

**Algorithm 1** MMMvII($\mathbf{G} = (\mathbf{V}, \mathbf{E})$, $\alpha$)

maxMatches $= \emptyset$
maxMatchSize $= 0$
$\delta = (1 + \alpha)/(1 - \alpha)$
**for all** $v_i \in \mathbf{V}$ **do**
  {Build list of $v_i$'s neighbours sorted by RPD of their edge to $v_i$}
  $\mathbf{L} = \{l_a \in \mathbf{V}\colon (v_i, l_a) \in \mathbf{E}, R(v_i)(l_a) \leq R(v_i)(l_{a+1})\}$
  **for** $a = 1$ **to** $|\mathbf{L}|$ **do**
    $v_j = l_a$
    **if** $j < i$ **then** {Avoids visiting an edge twice}
      **next** a
    **end if**
    $e_{min} = (v_i, v_j)$ {Assume an edge of minimum RPD}
    $\mathbf{H} = (\mathbf{U}, \mathbf{F}) = (\emptyset, \emptyset)$
    {Build vertex set $\mathbf{U}$ of subgraph $\mathbf{H}$}
    **for** $b = a + 1$ **to** $|\mathbf{L}|$ **do** {For all vertices ahead of $v_j$ in list $\mathbf{L}$}
      $v_k = l_b$
      $e_{ik} = (v_i, v_k)$
      $e_{jk} = (v_j, v_k)$
      **if** $R(e_{ik}) \geq R(e_{min}) \cdot \delta$ **then**
        {If $e_{ik}$ is not compatible with $e_{min}$, then this and all further $v_k$ are guaranteed to fail checks}
        **break**
      **else if** $R(e_{jk}) = R(e_{min})$ **and** $k < i$ **then**
        **next** b {Avoids duplicate results}
      **else if** $R(e_{min}) \leq R(e_{jk}) \leq R(e_{min}) \cdot \delta$ **then**
        {Both $e_{jk}$ and $e_{ik}$ are forward-compatible with $e_{min}$ – include $v_k$ in $\mathbf{U}$}
        $\mathbf{U} = \mathbf{U} \cup \{v_k\}$
      **end if**
    **end for**
    {Build edge set $\mathbf{F}$ of subgraph $\mathbf{H}$}
    **for all** $e_{xy} = (v_x, v_y) \in \mathbf{U} \times \mathbf{U}$ with $y > x$ **do** {For all pairs of vertices in $\mathbf{U}$}
      **if** $R(e_{xy}) = R(e_{min})$ **and** ($x < i$ **or** $y < i$) **then**
        **next** $e_{xy}$
      **else if** $R(e_{min}) \leq R(e_{xy}) \leq R(e_{min}) \cdot \delta$ **then**
        $\mathbf{F} = \mathbf{F} \cup \{e_{xy}\}$
      **end if**
    **end for**
    $\mathbf{M} = \text{maxCliques}(\mathbf{H})$ {Maximum cliques of $\mathbf{H}$ are its largest matches}
    **for all** $m \in \mathbf{M}$ **do**
      $m = m \cup \{v_i, v_j\}$
      **if** $|m| >$ maxMatchSize **then** {Keep only the globally largest matches}
        maxMatchSize $= |m|$
        maxMatches $= \{m\}$
      **else if** $|m| =$ maxMatchSize **then**
        maxMatches $=$ maxMatches $\cup \{m\}$
      **end if**
    **end for**
  **end for**
**end for**
**return** {maxMatches, maxMatchSize}

**Procedure 1** Ostergard($G = (V, E)$)

1: $\omega \leftarrow 0$ // Initialize max clique size
2: $Q \leftarrow \emptyset$ // Initialize set of max cliques
3: **for** $i = |V|$ **to** 1 **do** // Go through vertices in reverse order
4: $\quad U \leftarrow \emptyset$ // Build a list of $v_i$'s neighbours
5: $\quad$ **for** $j = i + 1$ **to** $|V|$ **do**
6: $\quad\quad$ **if** $(v_i, v_j) \in E$ **then**
7: $\quad\quad\quad U \leftarrow U \cup \{v_j\}$
8: $\quad\quad$ **end if**
9: $\quad$ **end for**
10: $\quad$ OstergardRecursive($\{v_i\}$, U) // Recursively expand the clique
11: $\quad c[v_i] \leftarrow \omega$ // Max clique size for subproblem starting at $v_i$
12: **end for**
13: **return** $\{Q, \omega\}$

**Procedure 2** OstergardRecursive($q$, U)

1: **if** U $= \emptyset$ **then**
2:  **if** $|q| > \omega$ **then** // Found new largest clique?
3:  $\omega \leftarrow |q|$
4:  Q $\leftarrow \{q\}$
5:  **else if** $|q| = \omega$ **then** // Modification to record *all* max cliques
6:  Q $\leftarrow$ Q $\cup \{q\}$
7:  **end if**
8: **else**
9:  **while** U $\neq \emptyset$ **do**
10:  **if** $|q| +$ |U| $< \omega$ **then** // Bound based on remaining vertices
11:  **return**
12:  **end if**
13:  $i \leftarrow \min\{j : u_j \in \text{U}\}$
14:  **if** $|q| +$ c$[u_i] < \omega$ **then** // Bound based on previous subproblems
15:  **return**
16:  **end if**
17:  U $\leftarrow$ U $\setminus \{u_i\}$
18:  U$' \leftarrow \{u' \in \text{U} : (u_i, u') \in \text{E}\}$
19:  OstergardRecursive($q \cup \{u_i\}$, U$'$)
20:  // Unmodified Ostergard also **return**s here if Line 2 was true in this or any child recursive iterations. Since we must find all max cliques, this early exit does not apply.
21:  **end while**
22: **end if**
